# Biased birth sex ratios of mammals and birds in zoos

**DOI:** 10.1038/s41598-025-05039-4

**Published:** 2025-07-01

**Authors:** Oscar G. Miranda, Fernando Colchero, José O. Valdebenito, Diego Cortez, Dalia A. Conde, Ivett Pipoly, András Liker, Balázs Vági, Mads F. Bertelsen, Albus Kilili, Araxi O. Urrutia, Tamás Székely

**Affiliations:** 1 HUN-REN-DE Reproductive Strategies Research Group, Department of Evolutionary Zoology and Human Biology, University of Debrecen, Debrecen, Hungary; 2 Biodiversity, Climate Change and Water Management Competence Centre, University of Debrecen, Debrecen, Hungary; 3 Department of Primate Behavior and Evolution, Max Planck Institute for Evolutionary Anthropology, Leipzig, Germany; 4 Department of Mathematics and Computer Science, University of Southern Denmark, Odense, Denmark; 5 Facultad de Medicina Veterinaria y Agronomía, Universidad de Las Américas, Campus Concepción, Concepción, Chile; 6 Instituto Milenio Biodiversidad de Ecosistemas Antárticos y Subantárticos (BASE), Santiago, Chile; 7 Centro de Ciencias Genómicas, Universidad Nacional Autónoma de México, Morelos, México; 8 Department of Biology, University of Southern Denmark, Odense, Denmark; 9 Behavioural Ecology Research Group, Center for Natural Sciences, University of Pannonia, Veszprém, Hungary; 10 HUN-REN-PE Evolutionary Ecology Research Group, University of Pannonia, Veszprém, Hungary; 11 Department of Ethology, Eötvös Loránd University, Pázmány Péter sétány 1/C, Budapest, H-1117 Hungary; 12 Copenhagen Zoo, Frederiksberg, Denmark; 13 Department of Life Sciences, Milner Centre for Evolution, University of Bath, Bath, UK; 14 Instituto de Ecologia, Universidad Nacional Autónoma de México, Ciudad de Mexico, Mexico; 15 Present Address: Departamento de Zoología, Universidad de Concepción, Concepción, Chile; 16 Department of Biology and Biochemistry, Milner Centre for Evolution, University of Bath, Bath, BA2 7AY UK

**Keywords:** Sex allocation, Mating systems, Evolutionary ecology, Sexual dimorphism, Biodiversity, Conservation, Zoo management, Conservation biology, Evolutionary ecology

## Abstract

Birth sex ratio biases can amplify extinction risks, especially in small, zoo-maintained populations which is of particular concern in species under threat of extinction. Thus, understanding the drivers of such biases is critical for conservation outcomes. We analysed birth records from 129 avian and 324 mammalian species in zoos worldwide between 1980 and 2021. Using Bayesian phylogenetic models, we found a phylogenetic signal in birth sex ratios (BSR), with substantial variation across clades. Penguins, falcons, and parrots showed slightly male-biased BSRs; ungulates showed female-biased BSR, and primates male-biased BSR. Across birds, variation in BSRs was predicted by sexual size dimorphism and clutch size, whereas in mammals, mating system was the main predictor of BSR. We identified 30 conservation flagship species with significantly biased BSRs, raising concern for the demographic sustainability of their captive populations. These results highlight the role of both evolutionary history and life-history traits in shaping sex ratio variation across taxa. Our findings underscore the importance of integrating phylogenetic and biological predictors into conservation planning and breeding program design. They also call for further research into the biological and management processes—that include sexual selection, parental investment, housing, and sexing practices—that may contribute to sex ratio variation in zoo populations.

**Supplementary Information:**

The online version contains supplementary material available at 10.1038/s41598-025-05039-4.

## Introduction

Biases in population sex ratios can dramatically increase the risk of extinction^1,2^. When sex ratios deviate from those that promote population stability—specific to each species’ breeding system—genetic diversity can decline, increasing the likelihood of inbreeding and compromising long-term viability^3–5^. Such imbalances also reduce the effective population size, making small populations particularly vulnerable to demographic collapse^6^.

Zoos play a crucial role in species conservation, yet zoo-maintained populations often remain critically small, with many falling below viability thresholds^7,8^. In species under threat of extinction where zoo populations represent an important component of remaining populations, even slight deviations in sex ratios can amplify the extinction risks^9^. Consequently, identifying birth sex ratio (BSR) biases in zoo populations and understanding the drivers underlying these imbalances is vital for conservation efforts^10^. Biased sex ratios can shape individual behavior, disease dynamics, and population stability^11^. For instance, an excess of males often results in heightened aggression, constant conflicts, and disrupted social dynamics^12,13^. Additionally, sex-specific disease prevalence may emerge under skewed ratios^14^impacting welfare and long-term population health.

Captive breeding environments differ fundamentally from wild ones in ways that may shape sex allocation. Unlike wild populations, zoo animals generally have reliable access to food, drinking water and medical care, minimizing resource limitation and poor health that are key ecological factors often linked to sex ratio variation^15^. On the other hand, captivity introduces its own challenges: restricted space, frequent interactions with humans, and artificial social groupings may all act as stressors^16^. These conditions differ from natural environments but are shared across institutions, offering a standardized context for large-scale analyses^17^. While breeding programs strive to optimize conditions, sex ratio biases may still emerge—not only as a byproduct of mortality or mismanagement^18,19^but because BSR itself can be inherently biased by genetic, environmental or the combination of genetic x environmental factors.

Sex allocation theory provides the conceptual framework to interpret these biases^20,21^. The Fisherian model of sex allocation predicts a 1:1 investment in sons and daughters when the reproductive value is equal^22,23^. However, the Trivers-Willard hypothesis propose that parents in good condition should favor the sex with higher potential reproductive returns^24^. This is especially relevant in polygynous systems, where a few males often monopolize reproduction, leading to high variance in male reproductive success^25,26^. In such contexts, producing sons may offer greater fitness payoffs for high-condition parents, while low-condition parents may gain more by producing daughters. Similarly, theories of local resource competition and enhancement suggest biases may emerge depending on the offspring’s dispersal and cooperative behavior^27^. Additionally, sexual size dimorphism can shape sex allocation, as producing the larger sex can be more costly for the parents^15^. Some theories also predict that the incentive to bias sex allocation may be diluted in species with larger broods^28,29^. Nonetheless, these models operate within the constraints imposed by genetic sex determination systems. For instance, birds and mammals differ in which sex is heterogametic—females in birds (ZW), males in mammals (XY)—and this distinction may shape how life-history traits influence sex allocation. Female-heterogametic systems tend to show more male-biased adult sex ratios than male-heterogametic ones, which might be partly due to reduced maternal control over offspring sex^30^. Comparative studies should therefore include taxa with diverse sex determination systems to assess whether theoretical predictors operate consistently across lineages.

Early large-scale comparative studies examined BSRs across birds and mammals from a demographic perspective but lacked phylogenetic context^31,32^. The first zoo-specific analysis raised concerns about demographic distortions in captive populations^33^and was later followed by studies testing predictors of BSR biases such as mating system and group size^34,35^. Some studies combined wild and captive datasets^36,37^ though only one of them focused exclusively on wild birds and found no general BSR bias^38^. Analyzing zoo bird data, Machado and Miller^39^ documented sex ratio skews across species, albeit they did not account for phylogeny or life-history correlates. More recent work has incorporated phylogenetic frameworks and life-history traits as predictors to explain variation in BSRs across taxa^40,41^. For instance, recent analysis in birds found male-biased BSRs to be more common ex-situ than in the wild, though ratios tended to approach parity at the global level; most variation occurred across institutions rather than across threat categories or geographic range^42^. Despite these advances, there is still a need of a comprehensive synthesis focused on zoo-held populations of both birds and mammals that accounts for phylogeny, investigate temporal shifts in BSR that may indicate biases due to animal husbandry, and integrates ecological and evolutionary predictors in the analyses.

Here we present the most extensive comparative analysis to date of BSRs in any taxa using birds and mammals held in zoos. We analyzed over 2.6 million birth records—approximately 460,000 from 129 avian species and 2.15 million from 324 mammalian species—collected from zoos worldwide over a 40-year period. Using these data, we test whether BSR biases are associated with specific phylogenetic lineages and key life-history traits, including sexual size dimorphism, mating system, and brood size. We also evaluate whether species with biased BSRs are disproportionately represented among higher IUCN threat categories (vulnerable or higher). Finally, we investigate temporal shifts in BSRs to identify species showing directional changes over time.

Importantly, these patterns will have direct implications for conservation management. Identifying consistent predictors of BSR bias can inform proactive, species-specific breeding strategies in zoos. Such insights may also support the development of tools to assess demographic vulnerability in species with limited reproductive data. This approach could be especially useful for rare or understudied taxa, helping optimize management decisions and improve the viability of captive populations.

## Results

### Birds

**Fig. 1:**
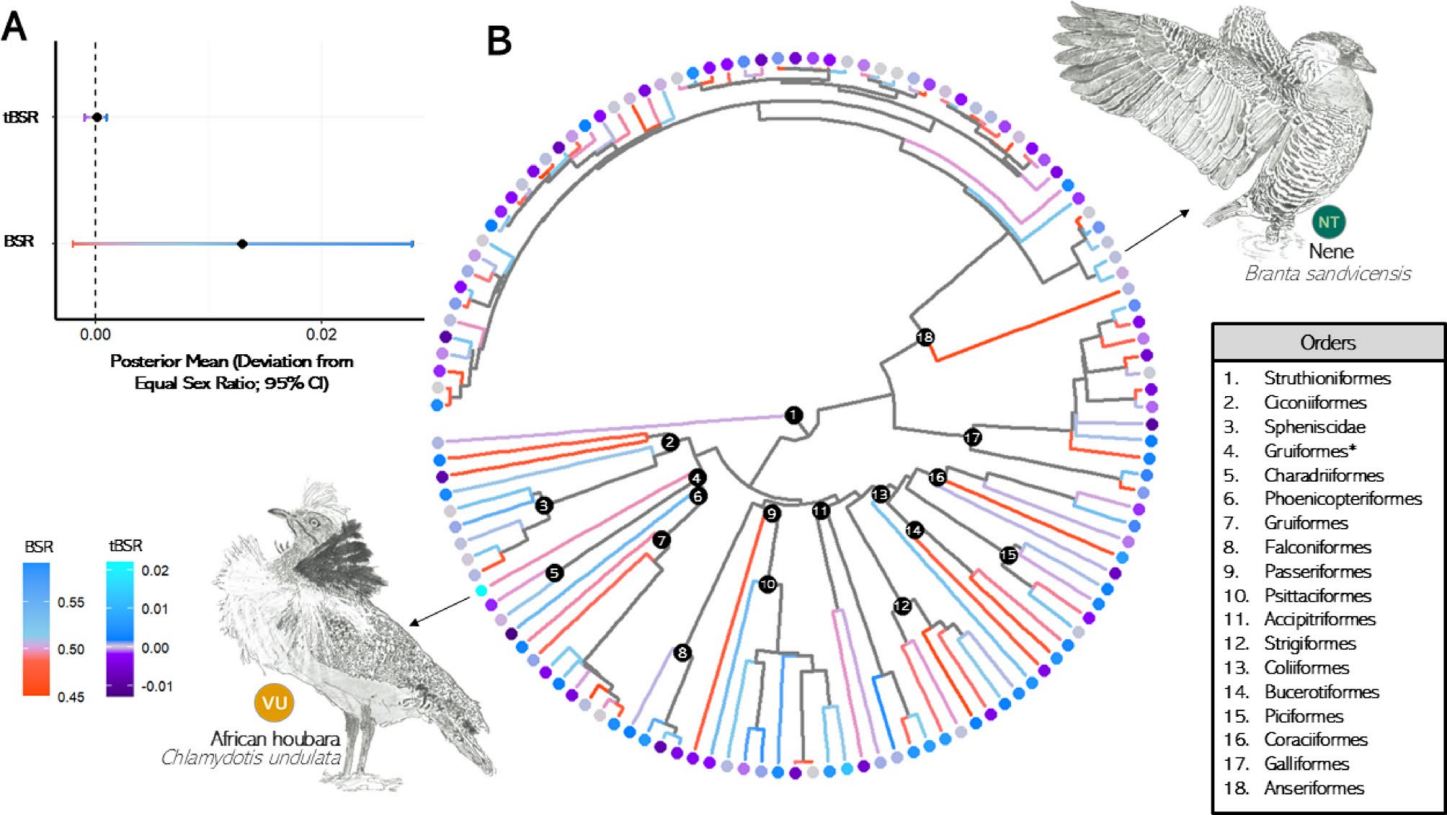
(**A**) Forest plot displaying posterior means (deviation from equal sex ratio) and their corresponding 95% credible intervals (CI) for birth sex ratio (BSR) and temporal trend in birth sex ratio (tBSR) in birds, using the intercept as an explanatory variable. Sex ratios are expressed as proportion of males. Posterior means and standard deviations were obtained from MCMCglm models. (**B**) Phylogenetic distribution of BSR and tBSR in 129 bird species. The plot shows a consensus tree constructed from 100 equally probable phylogenetic trees based on the bird phylogeny by Jetz et al.^70^using the least squares method. Branch color gradients represent BSR, with blue indicating male-biased and red indicating female-biased BSR. Tip point color gradients represent tBSR, with fluorescent blue indicating male-biased and purple indicating female-biased tBSR. Orders are numbered, indicating specific nodes from which they branch. The Gruiformes order is represented by nodes 4 and 7, as the houbara bustard (*Chlamydotis undulata*), a member of this order, branches before the most recent common ancestor (MRCA) of shorebirds, flamingos, and cranes. Two species are highlighted: the near threatened nēnē (*Branta sandvicensis*), endemic to the Hawaiian Islands, which showed a statistically significant male BSR (binomial test *p* = 0.049), and the vulnerable houbara bustard, known for its extravagant male courtship displays, which exhibited statistically significant tBSR (*β* = 0.021, *p* = 0.013).

Across 129 bird species, the overall (considering all years examined together) BSR was balanced, with no substantial deviation from parity. The 95% credible interval (−0.002 to 0.028) included 0 (posterior mean = 0.021, pMCMC = 0.013; Fig. 1A; Table S6) with moderate phylogenetic signal (λ = 0.341). Some groups, such as penguins, falcons, and parrots, exhibited a tendency toward male-biased BSR (Fig. 1B).

Examining individual species, the nēnē (*Branta sandvicensis*), a goose endemic to the Hawaiian Islands, exhibited a statistically significant bias towards females (0.399; Fig. 1B), with 1,823 male chicks out of 4,558 offspring (binomial test *p* = 0.049). BSR was statistically significantly male biased in mandarin duck (*Aix galericulata*), king penguin (*Aptenodytes patagonicus*), Harris’ hawk (*Parabuteo unicinctus*), and the superb parrot (*Polytelis swainsonii*). Importantly, no statistically significant BSR deviation was detected in threatened species (IUCN Vulnerable or higher).

The temporal trend in birth sex ratio (tBSR)—defined as the slope of the annual change in the proportion of male births—showed no substantial difference from zero, as indicated by the posterior mean close to zero and with a narrow 95% credible interval (−0.001 to 0.001; pMCMC = 0.030; Fig. 1A; Table S6). Whilst the tBSR exhibited a considerable phylogenetic signal (*λ* = 0.757), suggesting a phylogenetic effect on tBSR, this effect is likely biologically negligible given the narrow range of tBSR values (*β* in the range − 0.013 to 0.021). Although slope magnitudes depend on the scale of the time variable, the narrow range observed across species suggests that, regardless of scale, long-term directional changes in BSR are likely to be biologically minor. Only two species showed a statistically significant trend toward more males (i.e., more males in recent years): the houbara bustard (*Chlamydotis undulata*) classified as vulnerable by the IUCN (*β* = 0.021, *p* = 0.013; Fig. 1B), and the painted parakeet (*Pyrrhura picta*) (*β* = 0.013, *p* = 0.009).

### Mammals

**Fig. 2:**
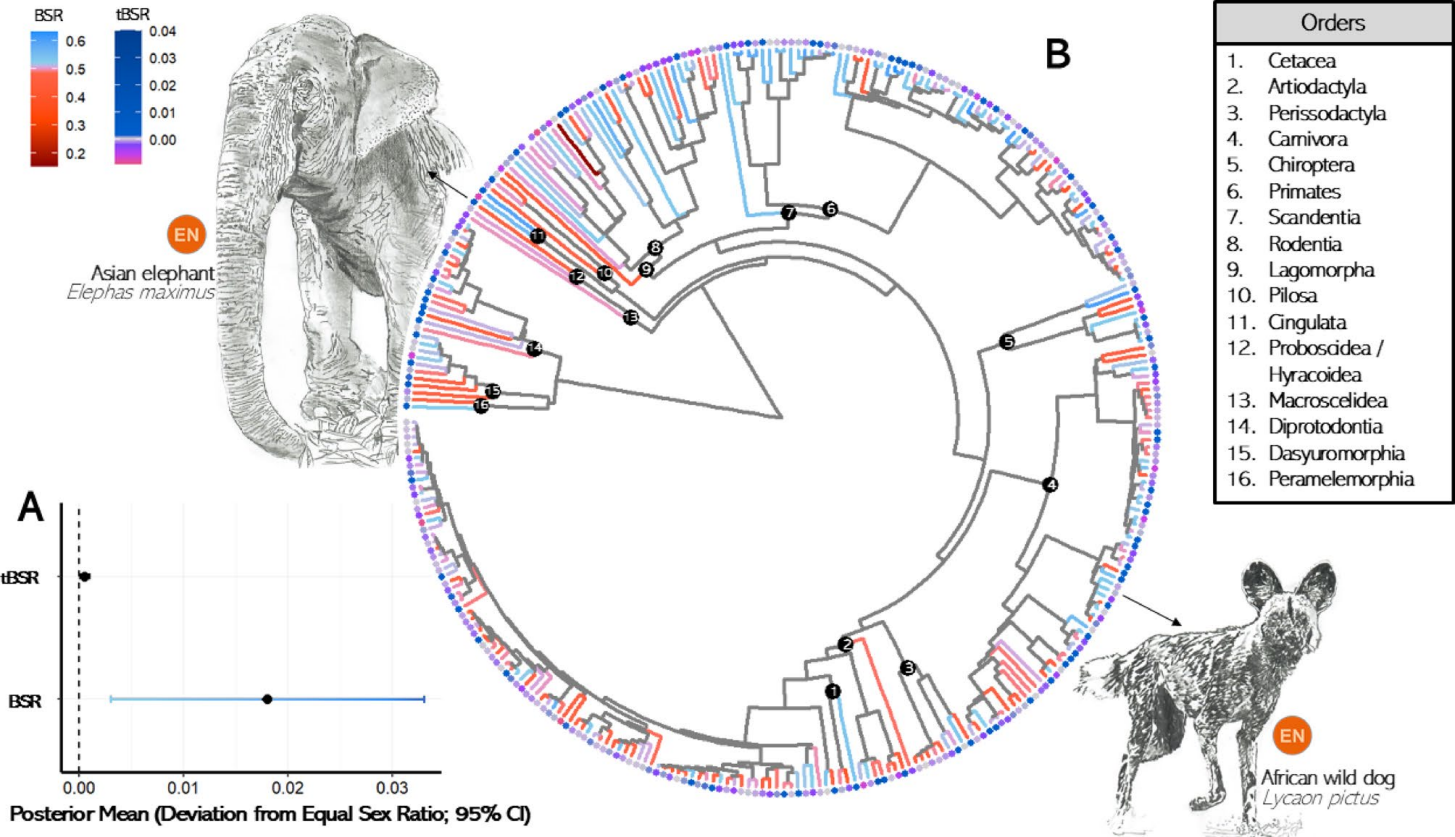
(**A**) Forest plot displaying posterior means (deviation from equal sex ratio) and their corresponding 95% credible intervals (CI) for birth sex ratio (BSR) and temporal trend in birth sex ratio (tBSR) in mammals, using the intercept as an explanatory variable. Sex ratios are expressed as proportion of males. Posterior means and standard deviations were obtained from MCMCglm models. (**B**) Phylogenetic distribution of BSR and tBSR in 324 mammal species. The plot shows a consensus tree constructed from 100 equally probable phylogenetic trees based on the mammal phylogeny by Upham et al. (2019), using the extend method. Branch color gradients represent BSR, with blue indicating male-biased and red indicating female-biased BSR. Tip point color gradients represent tBSR, with dark blue indicating male-biased and pink indicating female-biased tBSR. Orders are numbered, indicating specific nodes from which they branch. Two species are highlighted: the endangered African wild dog (*Lycaon pictus*), which showed a statistically significant male BSR (binomial test *p* = 0.022), and the endangered Asian elephant (*Elephas maximus*), which exhibited a statistically significant positive tBSR (*β* = 0.006, *p* = 0.009).

Across 324 mammal species, BSR was significantly male-biased, deviating positively from parity, with the observed sex ratio estimated at 0.505 and a 95% credible interval of [0.501, 0.508] (pMCMC = 0.006; Fig. 2A; Table S6). BSRs showed a strong phylogenetic signal (*λ* = 0.901), suggesting strong evolutionary influences on sex ratio adjustment mechanisms in mammals.

Visual inspection of the phylogenetic distribution (Fig. 2B) revealed that several clades tend to show consistent patterns. Female-biased BSRs appeared common in ungulates (Artiodactyla and Perissodactyla), most marsupial orders, and within the Carnivoran families Musteloidea and the genus *Panthera*. In contrast, male-biased BSRs were more frequently observed among Primates and the two Cingulata species (armadillos). Other major groups, including most of Carnivora outside Musteloidea, showed more balanced sex ratios. These clade-level patterns are descriptive, as no formal comparisons to higher taxonomic levels were conducted.

Examining individual species, 64 showed significant deviations from parity. Of these, 26 were female biased and 38 were male biased. Importantly, thirty threatened mammals exhibited a statistically significant BSR bias (Table 1), and several of these species are flagship conservation species, including the pygmy hippo (*Choeropsis liberiensis*), tiger (*Panthera tigris*), and Père David’s deer (*Elaphurus davidianus*) - which all showed a female-biased BSR. In contrast, the northern giraffe (*Giraffa camelopardalis*), African wild dog (*Lycaon pictus*), and ring-tailed lemur (*Lemur catta*) exhibited a male-biased BSR. Among threatened species: ungulates tended to show a female-biased BSR, while primates exhibited a male-biased BSR.

**Table 1:** Threatened mammals with statistically significant male- (or female-) biased birth sex ratios (BSR) and their IUCN status. Mammals classified as threatened (TH), endangered (EN), critically endangered (CR) and extinct in the wild (EW) were included.

Order	Scientific name	Common name	Birth sex ratio bias	IUCN status
Artiodactyla	*Choeropsis liberiensis*	Pygmy hippopotamus	Female	EN
Artiodactyla	*Elaphurus davidianus*	Père David’s deer	Female	EW
Artiodactyla	*Gazella arabica*	Arabian gazelle	Female	VU
Artiodactyla	*Gazella gazella*	Mountain gazelle	Female	EN
Artiodactyla	*Gazella subgutturosa*	Goitered gazelle	Female	VU
Artiodactyla	*Giraffa camelopardalis*	Northern giraffe	Male	VU
Artiodactyla	*Oryx leucoryx*	Arabian oryx	Female	VU
Artiodactyla	*Rusa timorensis*	Javan rusa	Female	VU
Carnivora	*Cuon alpinus*	Dhole	Male	EN
Carnivora	*Lycaon pictus*	African wild dog	Male	EN
Carnivora	*Panthera tigris*	Tiger	Female	EN
Chiroptera	*Pteropus rodricensis*	Rodrigues fruit bat	Male	EN
Perissodactyla	*Tapirus terrestris*	South American tapir	Male	VU
Primates	*Ateles fusciceps*	Black-headed spider monkey	Female	EN
Primates	*Callimico goeldii*	Goeldi’s marmoset	Male	VU
Primates	*Callithrix kuhlii*	Wied’s marmoset	Male	VU
Primates	*Cebuella pygmaea*	Western pygmy marmoset	Male	VU
Primates	*Cebus capucinus*	Colombian white-faced capuchin	Male	VU
Primates	*Eulemur albifrons*	White-headed lemur	Male	VU
Primates	*Eulemur coronatus*	Crowned lemur	Male	EN
Primates	*Eulemur fulvus*	Common brown lemur	Male	VU
Primates	*Lemur catta*	Ring-tailed lemur	Male	EN
Primates	*Leontopithecus rosalia*	Golden lion tamarin	Male	EN
Primates	*Nomascus leucogenys*	Northern white-cheeked gibbon	Male	CR
Primates	*Saguinus oedipus*	Cotton-top tamarin	Male	CR
Primates	*Sapajus xanthosternos*	Golden-bellied capuchin	Male	CR
Primates	*Trachypithecus auratus*	East Javan langur	Female	VU
Primates	*Varecia rubra*	Red ruffed lemur	Male	CR
Primates	*Varecia variegata*	Black-and-white ruffed lemur	Male	CR
Rodentia	*Marmota vancouverensis*	Vancouver Island marmot	Male	CR

Among mammals, tBSR showed no substantial deviation from zero, as indicated by the posterior mean (< 0.001) and the 95% credible interval (< 0.001 to 0.001; pMCMC = 0.010; Fig. 2A; Table S6). tBSR exhibited a weak phylogenetic signal (λ < 0.001), suggesting that species have not evolved sex ratio deviation mechanisms in response to long-term captive conditions. The only species showing a statistically significant tBSR deviation towards more males over time was the endangered Asian elephant (*β* = 0.006, *p* = 0.009; Fig. 2B).

### Predictors of BSR variation

**Fig. 3:**
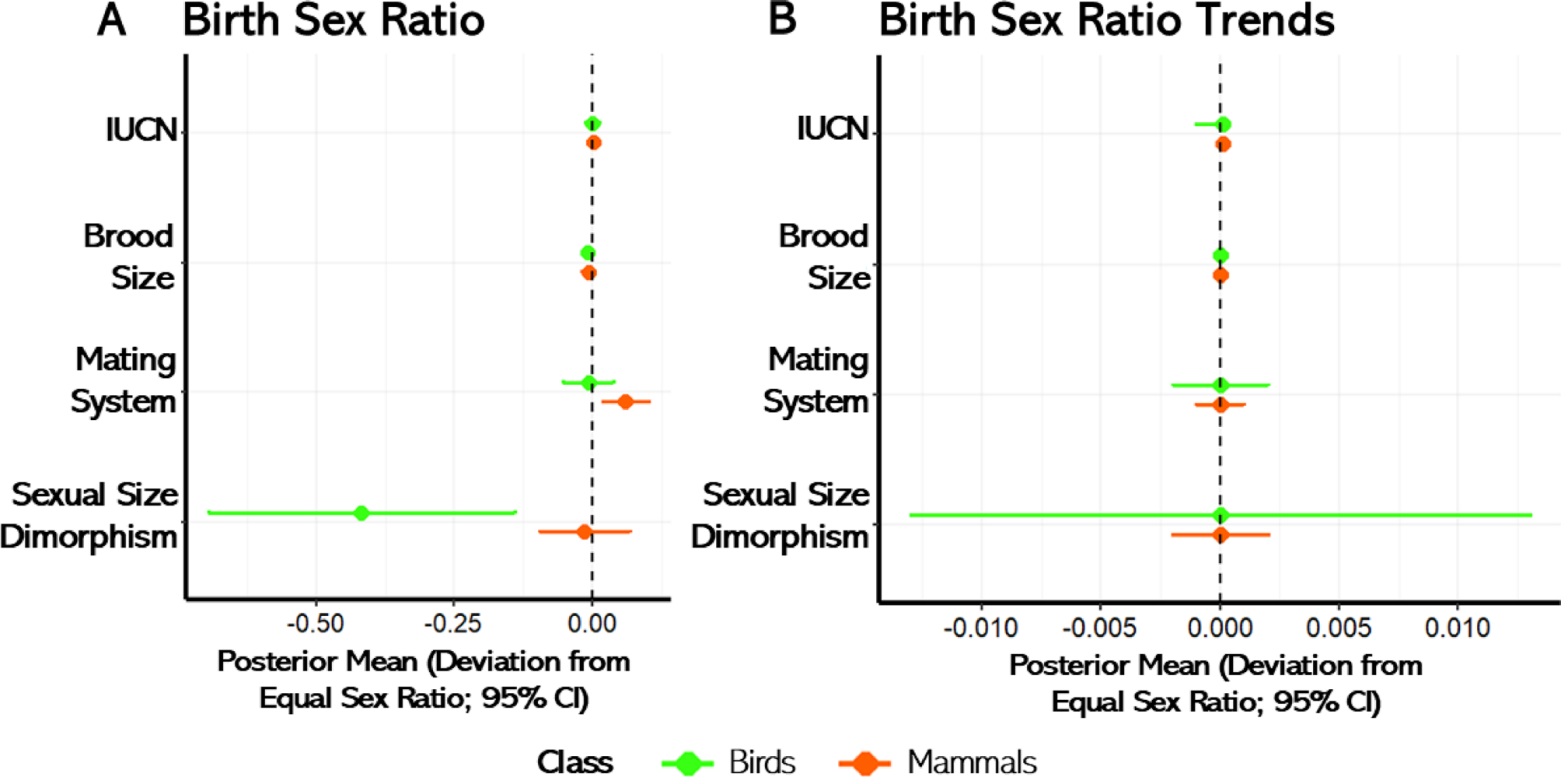
A forest plot of standardized posterior means and associated 95% credible intervals (CI) for the predictor variables used in models of (**A**) birth sex ratio (BSR) and in the (**B**) temporal trend in birth sex ratio (tBSR) in both birds and mammals (84 and 306 species, respectively). Posterior means and SDs were obtained from the MCMCglm models. Sample size were 129 bird species, and 322 mammal species for the model that include IUCN status as predictor.

For both birds and mammals, the relationships between BSR and three predictors—sexual size dimorphism, mating system, and brood size—showed distinct patterns across the models (Fig. 3; Table S7). In birds, biases in BSR were predicted by sexual size dimorphism and clutch size (Fig. 3A). Bird species with larger males compared to females exhibited a more biased female BSR (BSR = 0.397; logistic posterior mean = −0.418, 95% credible intervals [−0.695, −0.140], pMCMC = 0.012). Similarly, species with larger clutch sizes presented a more female biased BSR (BSR = 0.498; logistic posterior mean = −0.008, 95% credible intervals [−0.013, −0.002], pMCMC = 0.015). The model that best explained sex ratio variation in birds, based on the deviance information criterion (DIC), included sexual size dimorphism and brood size as additive factors.

For mammals, the model that best explained variation in BSR, based on the deviance information criterion (DIC), included mating system and brood size as additive predictors (Fig. 3A). In this model, monogamous species exhibited a more male-biased BSR (BSR = 0.519; posterior mean = 0.075, 95% credible interval [0.031, 0.119], pMCMC = 0.012). Brood size showed a negative effect on BSR in the same model (BSR = 0.497; posterior mean = −0.012, 95% credible interval [−0.023, −0.001], pMCMC = 0.072) suggesting that species with larger litters tended to have more female-biased birth sex ratios—but only when considered alongside mating system.

There were no significant life-history predictors of tBSR (Fig. 3B; Table S7). Lastly, the conservation status of species, as classified by the IUCN, did not predict variations in BSR or tBSR in either mammals or birds (Fig. 3; Table S8).

## Discussion

Biased birth sex ratios can have undermine the viability of *ex situ* populations, especially in conservation breeding programs. Our large-scale phylogenetic analysis revealed that while most bird species exhibited balanced BSRs, several clades—including penguins, falcons, and parrots—showed slight male biases. In mammals, we found stronger phylogenetic structuring, with ungulates exhibiting female-biased BSRs and primates showing male biases. Importantly, 30 conservation flagship mammal species showed significant BSR deviations, underscoring the need for targeted monitoring.

In birds, we found that sexual size dimorphism and clutch size predicted BSR variation. Species with larger males showed female-biased BSRs, possibly reflecting the higher energetic cost of producing sons^15,43^. Similarly, in species with larger broods, a female-biased BSR could arise as a strategy to maximize reproductive success, especially that in most birds females have shorter life-span than males^44^. These findings align with sex allocation theory, which suggests that life-history traits can influence the costs and benefits of producing each sex.

Nonetheless, significant deviations from parity were rare—only 5% of bird species showed biased BSRs. This supports the idea that random Mendelian sex allocation governs sex ratios in most cases^45^. Still, certain traits may predispose species to systematic biases. For example, the high proportion of waterfowl (~ 45%) in our dataset could have masked stronger patterns in other clades. Additionally, zoo practices like sexing only at maturity may obscure early biases, as postnatal mortality can equalize apparent ratios. These caveats warrant deeper investigation of sex allocation mechanisms in birds, especially in species with pronounced SSD or high clutch sizes.

Our results also echo previous findings that parrots, penguins, and geese may be particularly prone to BSR biases in captivity^39,46,47^. Although some of these species are colonial in the wild, their housing in dense zoo enclosures^48,49^—with altered social structures and limited space—could influence parental investment or stress responses, contributing to skewed sex ratios. Long-term parental care and social dynamics in captivity deserve closer attention as possible drivers of BSR variation.

In mammals, we observed a slight but statistically significant male bias in BSR, which may reflect a compensatory mechanism for typically female-biased adult sex ratios^30^. In many species, higher female survival leads to skewed ASRs in adulthood, and producing more males at birth may balance the reproductive pool^19,44^.

Crucially, mating system emerged as the main predictor of BSR in mammals. Monogamous species showed more male-biased BSRs, possibly because maintaining a balanced operational sex ratio is critical for successful pairings^32,50^. In contrast, polygynous systems may exert weaker selection for male-biased ratios, since only a few males reproduce^51^. Our binary classification of mating systems (monogamous vs. non-monogamous) may have oversimplified these dynamics, and future studies should incorporate quantitative metrics like degree of polygyny^52^.

We also found a strong phylogenetic signal in mammalian BSRs, reflecting deep evolutionary constraints. The consistent female-biased BSRs in ungulates align with the local resource competition hypothesis^27^ which predicts bias toward the dispersing sex. Even in zoos, maternal investment strategies may retain traces of these evolutionary patterns^53–57^. However, the male bias seen in many primates is less straightforward. Dispersal patterns alone do not explain the variation^34,37,41^ suggesting other factors—genetic, physiological, or social—may be at play.

Among the 450 + species analyzed, only three showed a statistically significant increase in male birth sex ratio over time: the painted parakeet, houbara bustard, and Asian elephant (*Elephas maximus*). These trends merit further research, especially in *P. picta*, whose subspecies taxonomy remains unresolved^58^. In houbara bustards, male bias aligns with their highly polyandrous mating system^59^ and artificial insemination in captivity may amplify this bias by favoring high-sperm males^60^. Asian elephants also showed a growing male bias, concerning given their polygynous system where few males reproduce^61,62^. In this species, elevated male-male competition and stress may hinder social stability and reproduction^13,63^. Careful breeding management is thus needed to prevent further imbalance.

We found no association between IUCN conservation status and BSR or tBSR in birds and mammals, suggesting that biased BSRs are not directly linked to conservation status. However, flagship species like the pygmy hippo, tiger, and Père David’s deer (extinct in the wild) had a female-biased BSR, while species such as the northern giraffe, African wild dog, and ring-tailed lemur exhibited a male-biased BSR. It is important to note, however, that our inclusion criteria required a minimum number of zoo-born individuals, which may have unintentionally biased our sample toward species with higher reproductive rates in captivity. Several highly threatened species have small population sizes and low intrinsic reproductive rates — traits that contribute both to their IUCN threat status and to lower birth numbers in captivity.

Moving forward, we recommend that breeding programs for species with known BSR biases—particularly those with large clutches, pronounced sexual size dimorphism, or monogamous systems—integrate BSR monitoring into management plans. In long-lived species, managers should also consider parental age, reproductive lifespan, and interbirth intervals as potential contributors to BSR biases. For example, age-related effects on BSRs are well documented in marsupials but remain underexplored in most zoo species^64^.

We recognize that our filtering thresholds—minimum number of births, proportion sexed, and ≥ 10 years of data—may have excluded short-lived or less-monitored species that could still exhibit BSR deviations. While relaxing these thresholds might uncover additional patterns, such results should be interpreted cautiously. For example, reptiles and amphibians, which we excluded, pose additional challenges due to temperature-dependent sex determination and sex reversal, but merit species-specific investigations given their high conservation concern. Moreover, some zoos initiate breeding programs with few founders and already skewed sex ratios. While inter-zoo collaboration can help rebalance demographics, it also underscores the importance of viewing zoos as interconnected metapopulations. Still, strong BSR biases at the species level demand careful management—especially in breed-for-release programs, where balanced sex ratios are critical for success. Interventions may include selective pairing, sex-specific resource provisioning, or breeding control to prevent further imbalance.

Birth sex ratios are more than a demographic parameter—they represent a fundamental axis of demographic stability or collapse. If left unmanaged, persistent BSR biases could silently erode the viability of species bred for conservation. Therefore, integrating sex ratio considerations into breeding strategies is not just prudent—it is imperative for the long-term success of conservation efforts.

## Materials and methods

### Zoo data extraction

We extracted data on the total number of male and female births in bird and mammal species from over 1,000 zoos worldwide. The data, spanning the period from 1980 to 2021, originated from the standardized Zoological Information Management Software (ZIMS), hosted by the international non-profit Species360^17,65^.

Because ZIMS integrates data across a global network of institutions, it reflects species-level demographic patterns rather than the decisions of any single zoo. This structure reduces the risk that our findings are biased by localized management practices. Even if a particular zoo phases out a species due to demographic concerns, cooperative breeding programs routinely redistribute individuals between institutions to balance population sex ratios. As such, exclusion of species solely due to BSR bias is unlikely to affect our dataset systematically. Notably, species such as the African bush elephant (*Loxodonta africana*), which exhibit known BSR biases in the wild, are still well represented in zoo collections, supporting the notion that such species are retained.

Next, we matched ZIMS species names with those in the VertLife database (https://vertlife.org/phylosubsets/). This step involved removing species without phylogenetic data, merging subspecies with species, and renaming species to synonyms (full list on Table S1). We identified synonyms using the ‘synonyms’ function in the taxize R package^66^ or through manual verification on the Integrated Taxonomic Information System (itis.gov).

### Species exclusion and selection criteria

To ensure a robust analysis, we applied several criteria to refine our dataset. First, we excluded domestic species as their BSR may have adapted to captivity^31,32,67^. We classified them as domestic if they are used for livestock or kept as pets (including exotic pets). Since ZIMS does not distinguish between wild and domestic species, we identified livestock as species that appeared in both our ZIMS database and Scherf^68^ and pets, including exotic pets, as species that appeared in both our database and Bays et al.^69^ (Table S2). These references were selected because they are widely recognized and provide comprehensive lists of species commonly categorized as livestock or pets, although no universally accepted criteria exist for defining domestic species.

To reduce biases emerging from small sample sizes, we excluded species with insufficient data and retained only those with at least 373 individuals born in zoos, representing the upper quartile for birds and mammals. Furthermore, we included only species where at least 69% of offspring were sexed (the median for birds and mammals) to minimize biases associated with unsexed individuals. A high number of unsexed individuals can indicate inconsistencies in sexing processes, which often depend on reproductive age or observable events. While detailed sexing methods for all species were not available, it is likely that sexually linked morphological traits, such as plumage differences or reproductive organs, were used in sexually dimorphic species, whereas molecular techniques were probably employed for monomorphic species (Bertelsen, pers. comm.).

Lastly, we excluded species with fewer than 10 years of data, as reliable time-series regression models typically require a minimum of 10 data points to ensure robust statistical analyses. As a result of these refinements, the final dataset included 129 bird species and 324 mammal species, with a mean of 1,665 sexed individuals per species (range: 273–16,503).

### Phylogenetic analyses

We accounted for variability in tree topology and branch lengths, by selecting 100 equally likely phylogenetic trees from the bird phylogeny by Jetz et al.^70^ and the mammal phylogeny by Upham et al.^71^ (vertlife.org). Importantly, all trees were coerced to be ultrametric using ‘force.ultrametric’ function from the phytools R package^72^.

We conducted all phylogenetic analyses separately for birds and mammals, repeated across one hundred phylogenetic trees. We averaged the outcomes across these trees and quantified phylogenetic variance as the standard deviation of the estimates.

### Phylogenetic signal in birth sex ratio

Before assessing whether BSR deviates from parity across species, we calculated its phylogenetic signal by means of Pagel’s lambda *λ* using the ‘phylosig’ function^72,73^. Pagel’s lambda was chosen because it provides a measure of the strength of the covariation in BSR as a function of the phylogenetic distance between species, aligning with our goal. Additionally, using the same ‘phylosig’ function we conducted a hypothesis test to determine whether the estimated λ was significantly different from 0 ^72^. Blomberg’s *K*, another phylogenetic signal estimate, was not used as we were not assessing whether BSR follows a Brownian motion model of evolution.

Specifically, BSR was defined as the ratio of male newborns relative to the total number of newborns (males and females) within a species. We treated BSR at the population level, considering all births of a species across zoos as belonging to a single population. This approach is justified since reproduction often involves individuals from different zoos, effectively linking them into a shared breeding population.

For birds, the results revealed a rather low phylogenetic signal (*λ* = 0.341, *p* = 0.017), which was statistically different from 0, but suggested modest influence of phylogeny on BSR (Table S3). Conversely, mammals exhibited a high phylogenetic signal (*λ* = 0.901, *p* < 0.000), indicating that phylogeny affects BSR in mammals (Table S3). Therefore, we accounted for phylogeny in analyses of both birds and mammals.

### MCMC generalized linear mixed models

To test whether BSR deviates from parity (1:1 male-to-female ratio), we fitted generalized linear mixed models (GLMMs) using Markov chain Monte Carlo (MCMC) methods with the MCMCglmm R package^74^. Specifically, we used male and female counts as the response (cbind(males, females) ~ 1), and incorporated phylogenetic structure through an inverse matrix (ginverse = list(species = Ainv_current_tree)).

We used a weakly informative inverse-Wishart prior with a variance (V) of 0.005 (prior = list(R = list(V = 0.005, nu = 0.05))), based on prior research showing animal population sex ratios are generally centered around 0.5. The family distribution was multinomial2, and the number of iterations, thinning, and burn-in were set to 50,000, 50, and 5,000, respectively, to ensure effective sample sizes above 200 for all 100 phylogenetic trees. Convergence was assessed using the Gelman-Rubin diagnostic^75^ implemented in the coda package^76^which confirmed that all chains had converged.

### Life-history traits as predictors

We explored the influence of three life-history traits (i.e. sexual size dimorphism social mating system and brood size) on BSR. Data for these traits were collected from published literature and online databases. SSD was calculated as log10(male body mass in grams/female body mass in grams) using sex-specific adult body masses^77^with positive values indicating heavier males and negative values indicating heavier females.

We classified social mating systems as monogamous or non-monogamous and recorded brood size as the average number of eggs or cubs per clutch or litter. Table S4 provides detailed references for the life history traits, which were based on wild populations unless otherwise noted.

We analyzed species with complete data in all three life-history traits (i.e., SSD, mating system brood size) resulting in 84 bird species and 306 mammal species. Using MCMCglmm, we modeled BSR variation with male and female counts as the response variable. We explored all predictor combinations, from single to multi-predictor models, in an additive framework. MCMCglmm prior, effective sample size and chain convergence were the same as previous models. We identified the best-fitting model using the deviance information criterion (DIC).

### Analysis of trends

We defined birth sex ratio trends (tBSR) as the change in BSR over time. For each species, we used the ‘tslm’ function from the forecast R package^78^ to fit a time-series linear model, with weighted sex ratio (ratio of males among sexed individuals per year) as the response and year as the predictor. We then analyzed the resulting slopes for phylogenetic signal and through MCMCglm models, with the family distribution being Gaussian, and a weaker prior (list(R = list(V = 0.005, nu = 0.002))), since there was no previous prediction on how strong is the variance of the population-level tBSR across animals.

### Identifying statistically significant birth sex ratio biases

To identify species with biased BSR, we performed binomial tests (binom.test(c(males, females), *p* = 0.5)) and adjusted p-values using the Benjamini-Hochberg^79^ method (p.adjust function). Species with statistically significant p-values were classified as biased. Additionally, species with statistically significant temporal trends were identified by slopes (*β*) differing from zero in the time-series linear model, with p-values similarly corrected.

### Exploring the role of IUCN status

Finally, we evaluated whether IUCN red list status predicted variations in BSR and tBSR. We retrieved conservation statuses from the IUCN website in November 2024 (Table S5), and after excluding two species due to insufficient data (*Dasyprocta azarae* and *Tragulus javanicus*), we converted IUCN status into an ordinal variable: Least Concern = 0, Near Threatened = 1, Vulnerable = 2, Endangered = 3, and Critically Endangered/Extinct in the Wild = 4. Using MCMCglm models, we fitted two models: one with male and female counts as the response and another with the time-series slopes as the response.

### Software and visualization

All analyses were conducted in R^80^. For visualization, tree plots were created using the ‘consensus.edges’ function with the least squares method^72^. Main figure components were produced using ggplot2 ^81^ and ggtree^82^with further modifications, including hand-drawn illustrations (drawn by O.G.M.), added in Microsoft PowerPoint.

### Ethical approval and data use statement

This study was conducted using data of animals in zoos and animals under human care, as authorized by Species360 research data use agreement #69,084. The use of these data was approved under the research request titled “Estimating sex ratios using Species360 data from tetrapods” and granted by Species360’s Board of Trustees. The data were obtained under the conditions set by Species360, which include restrictions on data sharing outside of the approved scientific study and proper acknowledgment of Species360 member institutions.

All methods in this study were performed in accordance with relevant guidelines and regulations as required by Species360’s research data use agreement. No direct experimentation on animals was conducted, and all data used were collected from pre-existing zoological records provided by Species360 member institutions.

## Electronic supplementary material

Below is the link to the electronic supplementary material.

Supplementary Material 1

## Data Availability

Raw data used to estimate brood sex ratio (Species360 Data Use Approval Number 69084) cannot be publicly shared, as Species360 is the custodian (not the owner) of their members’ data. Raw data are accessible through Research Request applications. Research Requests are reviewed by both the Species360 Research Committee and their Board of Trustees every four months. The Board of Trustees makes the final decision on data sharing, based on recommendations by the Research Committee. Once Species360 grants access to data, they are intended only for and restricted to use in the project they were approved for and for a single publication. The researcher cannot use them for other projects, publications and/or purposes, nor can the researcher share the data with third parties. Any further inquiries should be directed to support@species360.org.
