# Supplementary material for: Biased birth sex ratios of mammals and birds in zoos
Source: Sci Rep. 2025 Jul 1;15:20506. doi: 10.1038/s41598-025-05039-4 (PMC12219785; doi:10.1038/s41598-025-05039-4)
Supplement: Supplementary file 1 — Supplementary Material 1 [file 41598_2025_5039_MOESM1_ESM.docx]

Supplementary Information

**Biased Birth Sex Ratios of Mammals and Birds in Zoos**

Oscar G. Miranda, Fernando Colchero, José O. Valdebenito, Diego Cortez, Dalia A. Conde, Ivett Pipoly, András Liker, Balázs Vági, Mads F. Bertelsen, Albus Kilili, Araxi O. Urrutia & Tamás Székely
